# Is there evidence for factorial invariance of the COVID Stress Scales? an analysis of North American and cross-cultural populations

**DOI:** 10.3389/fpsyt.2024.1381124

**Published:** 2024-03-26

**Authors:** Blake A. E. Boehme, Laura Kinsman, Steven Taylor, Gordon J. G. Asmundson

**Affiliations:** ^1^ Anxiety and Illness Behaviours Laboratory, Department of Psychology, University of Regina, Regina, SK, Canada; ^2^ Department of Psychiatry, University of British Columbia, Vancouver, BC, Canada

**Keywords:** COVID stress scales, factorial invariance, confirmatory factor analysis, literature review, factor analysis

## Abstract

The COVID-19 pandemic impacted the mental health of more citizens globally than any previous modern viral outbreak. In response to the psychological challenges associated with COVID-19, the COVID Stress Scales (CSS) were developed to assess the presence and severity of COVID-related distress. The initial North American validation study of the CSS identified that the scale comprised five factors: danger and contamination fears, fear of socioeconomic consequences, xenophobia, checking and reassurance seeking, and traumatic stress symptoms. The CSS have since been validated across a multitude of international populations. However, findings support a five- and six-factor model. Methodological issues make interpreting most studies supporting a five-factor model challenging. The purpose of this study was to re-evaluate the factor structure of the CSS using data from North American samples, to assess for potential factorial invariance, and compare these results to cross-cultural findings. Multiple confirmatory factor analyses (mCFA) were conducted across 28 different groups (e.g., age, ethnicity/race, sex) from two large independent North American samples from 2020 (n = 6827) and 2021 (n = 5787), assessing the fit indices of the five-, six-, and alternative-factor model of the CSS. The current results provide evidence for factorial invariance of the six-factor model of the CSS across different North American demographics and highlight potential challenges in interpreting the results of studies that have supported a five-factor model of the CSS.

## Introduction

Since the beginning of the 21^st^ century, numerous pandemic, epidemic, and isolated infectious outbreak events have occurred. However, none of these previous modern contagion events have impacted the mental health of as many people globally as the COVID-19 pandemic. Previous pandemics, epidemics, and outbreaks have had unique characteristics related to the contagion; but, they share psychological factors and impacts, including increased anticipatory anxiety before a contagion has arrived in a specific location, the role of news media, increased prevalence of xenophobia and conspiracy theories, panic buying and hoarding, increased development and exacerbation of existing psychological disorders, and the spread of misinformation and dubious treatment protocols (1, 2). Considering the psychological impact of the COVID-19 pandemic (e.g., 3–6), it has been paramount to develop and validate psychological measures to understand its impact. The development of psychological measures is essential for assessing and treating distress and dysfunction in the context of COVID-19, and knowledge gleaned from this recent pandemic might help ameliorate the psychological impact of future pandemics (7, 8).

Fear and distress are common reactions to pandemics. Despite the availability of numerous measures of fear and distress in the behavioural sciences, pandemic-related distress is multifaceted and involves more than fear of contagion (1, 8). Therefore, specific measures of COVID-related distress have been developed to account for the relationships between pandemic-related factors that promote fear and distress during the COVID-19 pandemic. The COVID Stress Scales (CSS) are the most extensively validated measure of COVID-related distress. The CSS were developed to understand and measure COVID-related distress over the past week. Scale items were generated by consulting experts on health-related anxiety and reviewing extant literature on pandemics and epidemics (7, 8). Six primary psychological reactions to pandemics were identified: fear of infection, fear about coming into contact with potentially contaminated objects, worries about socioeconomic consequences, fear of others who might be infected, compulsive checking and reassurance-seeking, and traumatic stress (7, 8). Accordingly, the CSS was initially hypothesized to consist of six distinct scales designed to assess (1) danger fears (DAN), (2) contamination fears (CON), (3) fears about socioeconomic consequences (SEC), (4) xenophobia (XEN), (5) compulsive checking and reassurance-seeking (CHE), and (6) traumatic stress symptoms (TSS).

The original validation of the CSS comprised a web-based population-representative study conducted on a large sample of Americans and Canadians in March and April 2020. An exploratory factor analysis (EFA) conducted on the Canadian subsample of respondents and a confirmatory factor analysis (CFA) conducted on the American subsample of respondents endorsed a five-factor structure (7). As DAN and CON loaded onto a single factor in EFA, a five-factor model comprising DAN and CON combined, SEC, XEN, CHE, and TSS was retained instead of the initially hypothesized six-factor model. However, the CFA conducted in the original CSS validation study did not assess the fit indices of alternative models (e.g., six-factor), only assessing the fit indices of the EFA-derived five-factor model (7).

Studies evaluating the cross-cultural validity of the CSS have produced mixed findings concerning the factor model with the best fit (**Table 1**). There is evidence to support a five-factor model among Arabic, Greek, Persian, Polish, and Turkish populations (9, 11, 13, 14, 18). Alternatively, studies conducted among Chinese, Croatian, German, Hungarian, Nepali, Serbian, Spanish, Swedish, and Turkish populations have reported a better model fit for a six-factor model (12, 15–17, 19, 20). A study that assessed the factor structure and factorial invariance of the CSS across anxiety-related disorders found that the six-factor model was invariant (6). A challenge in assessing the validity of the factor analytic findings is that, of the five published studies that support the five-factor model, only one study (Polish) conducted a CFA on both the five- and six-factor model. There is also evidence from one study of alternative CSS models that some sub-scales other than DAN and CON may load on the same factor (e.g., TSS and CHE loading on the same factor in both a Dutch and a Polish sample; 14). Other variations in methodological procedures and sample size across studies may also contribute to differences in factor analytic findings. For example, two studies supporting a five-factor model performed an EFA and CFA on the same sample (9, 18).

**Table 1:** Results of published factor analytic studies of the COVID Stress Scales (CSS) across different populations.

Language	Author	Sample	EFA/CFA/Both	Supported Factor Structure	Were Five and Six Factor Assessed in CFA?	Notes
Arabic	(9)	Student (*N* = 1,080)	Both	Five-Factor	No	Instructions differed from that of the original scale. EFA and CFA conducted on same sample.
Chinese	(10)	General population (*N* = 2,116)	CFA	Six-Factor	Yes	
Croatian	(Franusic, 2021)	Primarily student sample (*N* = 638)	CFA	Six-Factor	Unknown	
English	Taylor et al. (7)	Population representative (*N* = 6,854)	Both	Five-Factor	No	
						
Greek	(11)	Convenience sample (*N* = 200)	EFA	Five-Factor	N/A	
Hungarian	(12)	Student sample (*N* = 350)	CFA	Six-Factor	Yes	
Persian	(13)	Anxiety disorders (*n* = 310) Obsessive-compulsive disorder (*n* = 300)	CFA	Five-Factor	No	
Polish	(14)	General population (*N* = 556)	Both	Five-Factor	Yes	
Serbian	(15)	General population (*N* = 961)	CFA	Six-Factor	Yes	
Spanish	(16)	Convenience sample (*N* = 1,424)	CFA	Six-Factor	Yes	
Swedish	(17)	Population representative (*N* = 3,044)	CFA	Six-Factor	Yes	
Turkish	(18)	Primarily student sample (*N* = 360)	Both	Five-Factor	No	EFA and CFA conducted on same sample.

EFA, exploratory factor analysis; CFA, confirmatory factor analysis. N/A, not applicable.

Considering the mixed findings supporting both a five and six-factor model of the CSS, the purpose of this study is twofold. First, we sought to determine which of various CSS factor solutions (i.e., five, six, or alternative factor models) best fit the data in two independent North American samples. Second, we assessed potential factorial invariance across sub-groups of respondents in these samples and how the results compare to cross-cultural studies.

## Methods

### Sample and data collection procedures

The data used in this study were collected from two independent North American samples at two different time points of the COVID-19 pandemic as part of the larger COVID Stress Study. Data from the first sample were collected in late winter-early spring 2020, and data from the second sample were collected in winter-early spring 2021. All respondents provided informed consent before completing the study, and data were collected using Qualtrics. The study was approved by the University of Regina Research Ethics Board (2020-043). A complete explanation of the sampling procedures and methodology of the COVID Stress Study is provided elsewhere (see 2, 7, 8).

The first sample (Sample 2020) comprised 6827 adults aged 18-94 years (*M* = 49.8 years, *SD* = 16.2), with 51.4% (3479) of the sample residing in Canada at the time of data collection. Sample 2020 was the sample used in the original validation studies of the CSS (7); however, the current analyses evaluate factor analytic models not considered in this initial validation study. The second sample (Sample 2021) comprised 5787 adults aged 18-92 years (*M* = 49.3 years, *SD* = 17.1), with 48.9% (2832) of the sample residing in Canada at the time of data collection.

Based on responses to demographic questionnaire items, respondents were assigned to groups based on their current country of residence, the period in which they were born, their sex, and their ethnicity. For example, if an individual respondent identified that they were currently residing in the United States, were born in 1977, are male, and are Asian, data would be included in the US, 1970-1979, male, and US Asian groups, respectively. Only those groups comprising ≥ 300 respondents were included in the final analyses based on the number of factors in the CSS (21, 22). A total of 14 groups were built, with seven groups from each sample (see **Table 2**). As there was no significant difference between the responses between sex and gender, nor enough respondents who identified as transgender, sex was chosen as the appropriate variable.

**Table 2:** Covid Stress Scales (CSS) model with best fit across North American groups.

		6	5	6	5	6	5	6	5	6	5
Group	Sample size	CFI		RMSEA 90% CI low		RMSEA		RMSEA 90% CI high		SRMR	
Sample 2020	6802										
US	3375	**0.94**	0.93	**0.052**	0.059	**0.053**	0.060	**0.054**	0.061	**0.038**	0.043
Canada	3479	**0.94**	0.93	**0.051**	0.056	**0.053**	0.057	**0.054**	0.058	**0.039**	0.041
US Asian	331	**0.91**	0.89	**0.064**	0.070	**0.068**	0.074	**0.073**	0.079	**0.055**	0.061
US White	1903	**0.93**	0.92	**0.055**	0.061	**0.057**	0.063	**0.059**	0.064	**0.042**	0.047
Canada Asian	352	**0.92**	0.91	**0.058**	0.061	**0.062**	0.065	**0.066**	0.069	**0.052**	0.055
Canada White	2748	**0.94**	0.92	**0.051**	0.055	**0.052**	0.057	**0.053**	0.058	**0.04**	0.042
Females	3216	**0.94**	0.93	**0.050**	0.055	**0.051**	0.057	**0.053**	0.058	**0.038**	0.041
Males	3638	**0.94**	0.92	**0.054**	0.059	**0.055**	0.060	**0.056**	0.061	**0.04**	0.043
1900-1949	667	**0.92**	0.90	**0.052**	0.058	**0.055**	0.061	**0.058**	0.064	**0.049**	0.054
1950-1959	1361	**0.93**	0.91	**0.050**	0.057	**0.052**	0.059	**0.054**	0.061	**0.044**	0.048
1960-1969	1302	**0.93**	0.91	**0.055**	0.060	**0.057**	0.062	**0.059**	0.064	**0.043**	0.047
1970-1979	1047	**0.94**	0.93	**0.053**	0.058	**0.056**	0.060	**0.058**	0.063	**0.040**	0.043
1980-1989	1166	**0.93**	0.92	**0.055**	0.060	**0.057**	0.063	**0.060**	0.065	**0.041**	0.044
1990-2002	978	**0.93**	0.92	**0.054**	0.057	**0.056**	0.059	**0.058**	0.062	**0.046**	0.048
Sample 2021	5787										
US	2955	**0.93**	0.89	**0.066**	0.081	**0.067**	0.082	**0.068**	0.084	**0.037**	0.050
Canada	2832	**0.93**	0.89	**0.064**	0.079	**0.065**	0.080	**0.067**	0.081	**0.04**	0.049
US Black	492	**0.91**	0.88	**0.069**	0.081	**0.072**	0.084	**0.075**	0.088	**0.045**	0.051
US White	1799	**0.93**	0.89	**0.069**	0.084	**0.070**	0.085	**0.072**	0.087	**0.041**	0.054
Canada Asian	327	**0.91**	0.86	**0.071**	0.088	**0.075**	0.092	**0.079**	0.097	**0.050**	0.058
Canada White	1931	**0.91**	0.87	**0.067**	0.081	**0.069**	0.083	**0.070**	0.085	**0.041**	0.052
Females	3313	**0.92**	0.89	**0.067**	0.082	**0.068**	0.083	**0.069**	0.084	**0.040**	0.049
Males	2491	**0.94**	0.90	**0.062**	0.077	**0.063**	0.079	**0.065**	0.080	**0.038**	0.049
1929-1949	572	**0.87**	0.80	**0.078**	0.095	**0.081**	0.098	**0.084**	0.101	**0.056**	0.070
1950-1959	1066	**0.89**	0.85	**0.075**	0.090	**0.077**	0.093	**0.080**	0.095	**0.046**	0.053
1960-1969	942	**0.91**	0.86	**0.068**	0.086	**0.070**	0.088	**0.073**	0.091	**0.043**	0.056
1970-1979	883	**0.92**	0.88	**0.069**	0.082	**0.072**	0.085	**0.074**	0.087	**0.044**	0.050
1980-1989	1246	**0.94**	0.91	**0.061**	0.074	**0.063**	0.076	**0.065**	0.078	**0.038**	0.046
1990-2003	1095	**0.93**	0.89	**0.061**	0.074	**0.063**	0.076	**0.065**	0.078	**0.040**	0.051

Years represent the time period respondents indicated their birth occurred when completing the Study: CFI, comparative fit index; RMSEA, root mean squared error of approximation; RMSEA CI, root mean squared error of approximation confidence interval; SRMR, standardized root mean squared residual. Figures that appear in bold indicate the superior fit score of each fit index for each group.

### Measures

Respondents provided demographic information, including their country of residence, sex, age, ethnicity, employment status, highest educational attainment, and household income. They also completed the CSS (7) as part of a larger battery of questionnaires. The CSS were designed to measure COVID-related distress over the past week and comprise 36 items distributed across six scales (i.e., DAN, CON, SEC, XEN, CHE, and TSS), each with six items. Items within the DAN, CON, SEC and XEN scales are scored on a 5-point Likert scale ranging from 0 (not at all) to 4 (extremely). The CHE and TSS items are scored on a 5-point Likert scale ranging from 0 (never) to 4 (almost always). High scores on the CSS indicate greater levels of COVID-related distress. The CSS has demonstrated good-to-excellent internal consistencies (7) and excellent validity and cross-cultural stability (9, 13, 15, 23). For the samples used in this current study, McDonald’s omega ranged from good to excellent in Sample 2020 for the individual scales (ω = 0.82 to ω = 0.94) and was excellent for the total score (ω = 0.96). For Sample 2021, McDonald’s omega (24) was excellent for the individual scales (ω = 0.89 to ω = 0.94) and the total scale (ω = 0.97). For estimating reliability, McDonald’s ω was used instead of Cronbach’s α due to the risk of overestimating reliability when using the latter (25). The range of values that are used to qualitatively represent reliability using ω are the same as those typically used for α; values in the range of.70-.80 indicate acceptable reliability,.80-.90 are good, and values greater than.90 are excellent.

### Statistical procedures

To assess for factorial invariance across groups (e.g., US, Canada, males, females, age), 56 mCFA were conducted. All analyses were conducted in R (version 4.2.2; 26), with mCFA conducted in the *lavaan* package (version 0.6-1.2; 27), and path diagrams plotted in the *lavaanPlot* package (version 0.6.2; 28). The estimation method for all CFA was robust maximum likelihood. The five-factor and six-factor models were assessed for best fit for each group. To explore potential alternative factor structures, a further 28 mCFA were conducted to assess the factor structure of a five-factor model that keeps DAN and CON as separate factors, combining TSS and CHE into a single factor (14). Goodness-of-fit was determined based on the assessment of the comparative fit index (CFI), root-mean-square error of approximation (RMSEA), including 90% confidence interval, and standardized root mean square residual (SRMR), based on empirically informed cut-off values, using maximum likelihood estimation (29, 30). Good fit was determined by CFI ≥ 0.90, whereas excellent fit was denoted by RMSEA ≤ 0.06, SRMR ≤ 0.08, and CFI ≥ 0.95. The CFI is used to compare the fit the specified model with the fit of a baseline null model which contains no relationships between variables (31).

## Results

The six-factor model demonstrated the best fit to the data, with evidence for factorial invariance across all groups assessed in this analysis (**Table 2**). The five-factor and six-factor models had adequate to excellent fit indices in Sample 2020, with the six-factor model consistently having marginally better measures of fit for this sample. For example, in the 2020 US sample, fit indices for the five-factor model were (CFI = 0.92, RMSEA = 0.060, SRMR = 0.043) compared to the six-factor model (CFI = 0.94, RMSEA = 0.053, SRMR = 0.038)

In Sample 2020, fit indices for the six-factor model ranged from (CFI = 0.91, RMSEA = 0.068, SRMR = 0.055) in the US Asian group to (CFI = 0.94, RMSEA = 0.051, SRMR = 0.038) in the Female group.

For Sample 2021, the five-factor model had poorer fit indices across all groups compared to the five-factor model in Sample 2020, with most fit indices bordering on adequate to good. Again, in Sample 2021, the six-factor model had better fit indices across all groups than the five-factor model, with most fit indices ranging from good to excellent. For example, fit indices for the five-factor model in the 2021 US sample were (CFI = 0.89, RMSEA = 0.082, SRMR = 0.050) compared to the six-factor model in the same US sample (CFI = 0.93, RMSE = 0.067, SRMR = 0.037). Fit indices for the six-factor model in Sample 2021 ranged from (CFI = 0.87, RMSEA = 0.081, SRMR = 0.056) in the born 1929-1949 group to (CFI = 0.94, RMSEA = 0.063, SRMR = 0.038) in the Male and people born 1980-1989 groups (both groups had identical fit

indices). The alternative five-factor model (CHE and TSS as a single factor) had significantly poorer fit indices than the six-factor and original five-factor models across all sub-groups.

**Figure 1:**
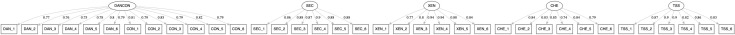
*Path diagram for the five-factor model of Covid Stress Scales in the 2021 US sample.* CHE, compulsive checking and reassurance seeking; DANCON, fears of danger and contamination; SEC, fears of socioeconomic consequences of the virus; TSS, traumatic stress symptoms; XEN, xenophobic fears. The numbers on the arrows represent factor loadings.

**Figure 2:**
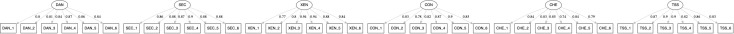
*Path diagram for the six-factor model of Covid Stress Scales in the 2021 US sample.* CHE, compulsive checking and reassurance seeking; CON, fears of contamination; DAN, danger fears; SEC, fears of socioeconomic consequences of the virus; TSS, traumatic stress symptoms; XEN, xenophobic fears. The numbers on the arrows represent factor loadings.

Assessment of confidence intervals, factor loadings, and residuals provided further evidence for factorial invariance and superior fit of the six-factor model. When assessing the 90% confidence intervals for RMSEA across samples, there was no overlap between the five and six-factor models. Factor loadings were typically higher for the DAN and CON factors when separated into two factors. Likewise, residuals were typically lower for DAN and CON items when separated into two factors. Path diagrams providing factor loadings are provided in **Figures 1**, **2**, depicting the five-factor and six-factor models from the US group in 2021, respectively. In the 2021 US group, factor loadings for DAN and CON items were consistently higher in the six-factor model. For many of the group analyses, the interfactor correlation was moderately high, especially between the TSS and CHE factors in both the five and six-factor models (0.74 in each model) and the DAN and CON factors (0.78) in the six-factor model. The correlations raised the need to test for discriminant validity between these factors.

To test for discriminant validity, the average variance extracted (AVE) was calculated for each factor for all the groups and was compared to the shared variance between factors across those groups using the *semTools* package (version 0.5-6; 32). Values of AVE > 0.50 are typically considered satisfactory, indicating that > 50% of the variance in a measure is due to the proposed construct (33). The shared variance between factors was calculated by squaring the correlation coefficient. To demonstrate discriminant validity, the AVE of a factor must be higher than the shared variance between that factor and any other factor. If the AVE of a factor exceeds the shared variance between factors, there is evidence that having two factors instead of one provides more information regarding the variation of items within factors than the amount of variation explained by any overlap between factors.

Comparing the AVE to the shared variance across five and six-factor models across all groups provided evidence for discriminant validity, with AVE being consistently higher than the shared variance between factors. For example, the interfactor correlation between DAN and CON and between TSS and CHE in the six-factor model of US respondents from 2021 were 0.78 and 0.74, respectively. Therefore, the shared variance of the DAN and CON factors was 0.61, while the shared variance between the TSS and CHE factors was 0.55. The AVE for the DAN, CON, TSS, and CHE factors from the 2021 US group were 0.70, 0.71, 0.75, and 0.66, respectively, exceeding the shared variance, and supporting discriminant validity between-factors. For comparison, the AVE when combining DAN and CON into a single factor in this same group was lower (0.63), a consistent finding across all groups.

## Discussion

The current study was designed to further evaluate the factor structure of the CSS, assess for potential factorial invariance in two independent North American samples in 2020 and 2021, and to compare these results with cross-cultural validation results. Although some prior studies have reported support for a five-factor model of the CSS (7, 9, 11, 14, 18), all but one of these studies (14) failed to conduct a CFA assessing fit indices for both the five and six-factor model. Other differences in methodological approach across cross-cultural studies may have also played a role in these mixed findings. A potential reason that many cross-cultural studies did not conduct a CFA on both the five and six-factor model of the CSS is that the original North American validation study only assessed the fit indices of the five-factor model in their CFA (7). The analyses presented in this study support the factorial invariance of the six-factor model of the CSS across different groups by demographics, including males and females, ethnic groups, and all age groups of respondents residing in the United States and Canada.

The limitations of assessing for potential factorial invariance based on prior studies come primarily from the fact that nearly half of these studies, and almost all those that support a five-factor model of the CSS, did not assess alternative models in their CFA. Another potential influence on varied results across previously published studies is potential issues in translating the CSS from English to another language. The primary limitation of the analyses presented in the current study is that many groups (e.g., Canadian Indigenous People) did not have enough respondents to be included in the mCFA. Considering that some of the groups make up a large portion of the population (5.0% of the Canadian population are Indigenous People; Statistics Canada, 2022) and, collectively, these groups make up a significant portion of the population, it will be important for future researchers to assess the validity of the CSS in these underrepresented groups.

The CSS are a measure of COVID-related distress which has been extensively studied and validated during the COVID-19 pandemic. Other modern pandemics have shared similar characteristics with the COVID-19 pandemic, with the primary difference being that the COVID-19 pandemic impacted more people globally. The CSS holds considerable potential to be adapted to assess and conceptualize pandemic-related stress in the context of future pandemics. The results of this study provide evidence that using total scores and separate scores from the originally conceptualized six scales of the CSS, with DAN and CON representing their own scales, may provide more accurate (invariant) and nuanced information regarding pandemic-related distress. Moving forward, researchers validating the CSS across different populations or in the context of future pandemics should use methodologies that assess for the validity of five and six-factor models of the CSS but also explore alternative models of pandemic-related distress that may exist in their population of study.

## Data availability statement

The data analyzed in this study is subject to the following licenses/restrictions: Currently not publicly available due to funding body. Requests to access these datasets should be directed to blake.boehme@uregina.ca.

## Ethics statement

The studies involving humans were approved by University of Regina Institutional Review Board. The studies were conducted in accordance with the local legislation and institutional requirements. Written informed consent for participation was not required from the participants or the participants’ legal guardians/next of kin in accordance with the national legislation and institutional requirements.

## Author contributions

BB: Conceptualization, Formal Analysis, Methodology, Software, Writing – original draft, Writing – review & editing. LK: Writing – original draft. ST: Data curation, Funding acquisition, Supervision, Writing – review & editing. GA: Conceptualization, Data curation, Funding acquisition, Supervision, Writing – review & editing.
